# Genes Associated with Retinitis Pigmentosa and Allied Diseases Are Frequently Mutated in the General Population

**DOI:** 10.1371/journal.pone.0041902

**Published:** 2012-07-27

**Authors:** Koji M. Nishiguchi, Carlo Rivolta

**Affiliations:** Department of Medical Genetics, University of Lausanne, Lausanne, Switzerland; Innsbruck Medical University, Austria

## Abstract

**Background:**

Retinitis pigmentosa and other hereditary retinal degenerations (HRD) are rare genetic diseases leading to progressive blindness. Recessive HRD are caused by mutations in more than 100 different genes. Laws of population genetics predict that, on a purely theoretical ground, such a high number of genes should translate into an extremely elevated frequency of unaffected carriers of mutations. In this study we estimate the proportion of these individuals within the general population, via the analyses of data from whole-genome sequencing.

**Methodology/Principal Findings:**

We screened complete and high-quality genome sequences from 46 control individuals from various world populations for HRD mutations, using bioinformatic tools developed in-house. All mutations detected in silico were validated by Sanger sequencing. We identified clear-cut, null recessive HRD mutations in 10 out of the 46 unaffected individuals analyzed (∼22%).

**Conclusions/Significance:**

Based on our data, approximately one in 4–5 individuals from the general population may be a carrier of null mutations that are responsible for HRD. This would be the highest mutation carrier frequency so far measured for a class of Mendelian disorders, especially considering that missenses and other forms of pathogenic changes were not included in our assessment. Among other things, our results indicate that the risk for a consanguineous couple of generating a child with a blinding disease is particularly high, compared to other genetic conditions.

## Introduction

Retinitis pigmentosa and allied diseases (collectively called hereditary retinal degenerations, or HRD) are a group of Mendelian disorders causing progressive degeneration of the light-sensing cells of our eyes, the photoreceptors. The majority of patients initially experience night blindness, usually during adolescence, followed by loss of peripheral vision in any lighting environment. At later stages, central vision can be lost as well, leading in many cases to legal or complete blindness [1].

Although dominant and X-linked HRD forms exist and account for many cases, most patients inherit this condition as a recessive trait, from parents who are healthy carriers of heterozygous mutations. Up to now, mutations in more than 100 distinct HRD genes have been described, making this pathology one of the most genetically heterogeneous human disease identified so far [2]. Considering that the overall prevalence of HRD is ∼1 in 3,500 individuals (∼1 in 6,000 for recessive HRD) [3], [4], the number of cases who can be attributed to mutations in any specific HRD gene is therefore extremely small.

According to the laws of population genetics and to common sense, frequent Mendelian diseases are determined by frequent mutations that are present within the general population, while rare diseases by rare mutations. HRD is relatively rare, however its elevated genetic heterogeneity may result in a high frequency of unaffected carriers of recessive mutations, if any of the many genes involved in the disease are considered. Thanks to the wealth of genomic information that is currently available, we have directly assessed such a frequency.

## Methods

### Ethics Statement

This study involved the use of fully anonymized, publicly-available DNA samples that were purchased from the biological repository at the National Institute of General Medical Sciences (NIGMS). Policies in force at NIGMS strictly prohibit the release of any information allowing the identification of the individuals from whom DNA samples were collected, making unnecessary additional local IRB approval [5].

### Samples

Since we needed to assess the frequency of HRD mutations per completed, individual genomes, we refrained from using data from on-line databases that contained partial genomic information or information based on uneven sequence coverage across the whole genome (e.g. the 1,000 Genomes database). Instead, we selected a smaller but high-quality dataset composed of genomes from 46 healthy and unrelated individuals from the NIGMS repository, available at http://www.completegenomics.com (Feb. 2011 release, assembly software version 1.10.0.26) [6]. The cohort analyzed included European Americans, African Americans, Mexicans, Italians, Maasai, Yoruba, Luhyas, Chinese, Japanese, and Gujaratis.

**Table 1:** Mutations in hereditary retinal degeneration genes identified in control subjects from various world populations.

NIGMS ID	Ethnicity	Mutated gene	DNA variation	Protein or splicing variation
NA10851	European American	*ROM1*	c.493_494insA	p.R165fs
NA19700	African American	*ROM1*	c.868delC	p.Q290fs
NA18526	Chinese	*PCDH15*	c.4866_4867insGACA	p.D1623fs
NA20846	Gujarati	*CEP290*	c.7392_7393delAG	p.E2465fs
NA20850	Gujarati	*CEP290*	c.4393C>T	p.R1465X
NA19020	Luhya	*PCDH15*	c.5264_5265insGTCT	p.Q1755fs
NA20509	Italian	*USH2A*	c.917_918insCAGC	p.S307fs
NA18501	Yoruba	*CC2D2A*	c.1017+1G>A	IVS11+1G>A
NA18504	Yoruba	*ABCA4*	c.834delT	p.S278fs
NA19129	Yoruba	*AHI1*	c.−55+1G>T	IVS2+1G>T

The NIGMS IDs of the individuals whose genome was analyzed were: NA06985, NA06994, NA07357, NA10851, NA12004, NA18501, NA18502, NA18504, NA18505, NA18508, NA18517, NA18526, NA18537, NA18555, NA18558, NA18940, NA18942, NA18947, NA18956, NA19017, NA19020, NA19025, NA19026, NA19129, NA19648, NA19649, NA19669, NA19670, NA19700, NA19701, NA19703, NA19704, NA19735, NA19834, NA20502, NA20509, NA20510, NA20511, NA20845, NA20846, NA20847, NA20850, NA21732, NA21733, NA21737, and NA21767.

### In Silico Screening for HRD Mutations

We first compiled a list of 106 genes that were previously associated with recessive syndromic and nonsyndromic hereditary retinal degeneration, based on the information provided in the Retinal Information Network (RetNet; https://sph.uth.tmc.edu/retnet/) and the Online Mendelian Inheritance in Man (www.ncbi.nlm.nih.gov/omim) websites (Table S1). By using simple text parsing Perl scripts (available on request), we then identified all DNA changes found to be present in these genes. Among these variants, we finally selected clear-cut deleterious mutations (nonsense, frameshift, or IVS+1, +2, −1, −2 splice site changes).

### Validation and Annotation of Identified Mutations

All deleterious mutations identified in silico were validated by direct Sanger sequencing on PCR-amplified DNA, using as genomic template DNA that was purchased from the NIGMS repository (http://ccr.coriell.org/nigms). The reference sequences used for variant annotation were: NM_000327.3 and NP_000318.1 for *ROM1*, NM_001142769.1 and NP_001136241.1 or NM_001142771.1 and NP_001136243.1 for *PCDH15*, NM_025114.3 and NP_079390.3 for *CEP290*, NM_206933.2 and NP_996816.2 for *USH2A*, NM_001080522.2 and NP_001073991.2 for *CC2D2A*, NM_000350.2 and NP_000341.2 for *ABCA4*, and NM_017651.4 and NP_060121.3 for *AHI1*. For the remaining HRD genes, all reference sequences enrolled in the NCBI database build 37.1 were used for annotation.

## Results

We identified clear-cut, null recessive mutations in 10 out of 46 fully-sequenced genomes from control samples, making the cumulative frequency of unaffected carriers of pathogenic HRD alleles ∼22% (95% confidence interval  =  10–34%, Table 1). All mutations were detected in a heterozygous state, as expected, and were verified by direct Sanger sequencing. No genome carried more than one HRD mutation.

None of these DNA changes were previously recognized as HRD mutations, with the exception of c.4393C>T (p.R1465X) in *CEP290*. This DNA variant, detected in a Gujarati (Indian) control, was originally described in individuals with Joubert syndrome–related disorders from Belgium, Brazil, and the United States [7].

## Discussion

Recent sequencing of personal genomes has shown that, out of the many non-synonymous variants identified [8], on average every individual is an unaffected carrier of 10–20 recessive alleles for Mendelian conditions [9], [10]. Our finding experimentally that 1 in 4–5 individuals from the general world population could be a carrier of mutations linked to hereditary blindness confirms and extends previous theoretical estimates on HRD genetic epidemiology [11]. Furthermore, our measurements represent a significant underestimation of the real frequency, considering that no mutations other than definite null changes were considered and that known HRD genes account for only 50–70% of the diagnosed cases [4]. According to the data reported in the Human Gene Mutation Database [12] for the 106 genes analyzed, the average ratio between null and missense mutations is 1 to 0.87. If we use this proportion to roughly extrapolate our findings and take missense mutations into consideration, the frequency of unaffected carriers would be 1 in 2.5 individuals. Further extrapolation to patients with HRD who are negative for mutations in known genes (assumed to represent 30% of all recessive cases, based on data from retinitis pigmentosa [4]) would lead to the even more dramatic figure of 1 unaffected carrier in 1.7 individuals.

The “aggregate frequency of mutations” of a genetically heterogeneous Mendelian disorder can show significant disparity with our knowledge of “disease frequency” or prevalence, which may be confusing at times. This is because the aggregate frequency is strongly influenced by the number of genes and percent of cases that can be attributed to each gene, which are unique to every genetic condition. Diseases like HRD, displaying high genetic heterogeneity and a relatively even contribution for each gene, show in general high aggregate frequency despite a low overall prevalence [11].

Such an elevated frequency of unaffected carriers of HRD mutations has fortunately a limited influence on the likelihood of generating affected offspring (i.e. 1/6,000, on average), since the chance for two unrelated parents to carry each a heterozygous mutation in the very same gene remains low. However, it has a few consequences for both genetic counseling and research, especially in our age of full genome or exome sequencing. First, it is likely that, in addition to causative mutations, other HRD alleles could be accidentally present in patients with retinal diseases. Misinterpretation of such findings could complicate molecular diagnoses or give rise to false speculations about oligogenic inheritance or dominant effects of recessive alleles. A second consequence is that variation databases from cohorts of healthy individuals, routinely used as negative controls for suspected HRD variants in molecular testing, may in fact contain true mutations.

Most important, a high frequency of HRD mutation carriers translates into a quite increased risk for a consanguineous couple of generating a child with a blinding disease. This phenomenon is particularly evident in populations displaying an elevated degree of inbreeding [13]–[16], for which the prevalence of hereditary blindness is higher than the average [17]. In such a context, any recessive mutations in any HRD genes that would be present in both parents could be easily brought to homozygosity in the offspring, without benefiting from the buffering effect of genetic heterogeneity.

## Supporting Information

Table S1 **HRD genes screened for pathogenic mutations.**
(DOC)
