# Supplementary material for: Genes Associated with Retinitis Pigmentosa and Allied Diseases Are Frequently Mutated in the General Population
Source: PLoS One. 2012 Jul 27;7(7):e41902. doi: 10.1371/journal.pone.0041902 (PMC3407128; doi:10.1371/journal.pone.0041902)
Supplement: Table S1 — HRD genes screened for pathogenic mutations. (DOC) [file pone.0041902.s001.doc]

**Table S1.**

*ABCA4*

*ABHD12*

*ADAM9*

*AGTPBP1*

*AHI1*

*AIPL1*

*ALDH3A2*

*ALMS1*

*ARL6*

*ATXN7*

*BBS1*

*BBS10*

*BBS12*

*BBS2*

*BBS4*

*BBS5*

*BBS7*

*BBS9*

*C2ORF71*

*CABP4*

*CC2D2A*

*CDH23*

*CEP290*

*CERKL*

*CLN3*

*CLN5*

*CLN6*

*CLN8*

*CLRN1*

*CNGA1*

*CNGB1*

*CNNM4*

*CRB1*

*CRX*

*CTNS*

*CYP4V2*

*DFNB31*

*DHDDS*

*ERCC2*

*ERCC6*

*ERCC8*

*EYS*

*FAM161A*

*FLVCR1*

*GNAT1*

*GPR98*

*GRK1*

*GUCY2D*

*HMX1*

*IDH3B*

*IMPG2*

*INPP5E*

*INVS*

*IQCB1*

*KCNV2*

*LCA5*

*LRAT*

*MERTK*

*MKKS*

*MTTP*

*MYO7A*

*NPHP1*

*NPHP3*

*NR2E3*

*NRL*

*OAT*

*PCDH15*

*PCDH21*

*PDE6A*

*PDE6B*

*PDE6G*

*PEX1*

*PEX12*

*PEX26*

*PEX5*

*PEX7*

*PHYH*

*PRCD*

*PROM1*

*PRPH2*

*PXMP3*

*RBP3*

*RBP4*

*RD3*

*RDH12*

*RGR*

*RHO*

*RLBP1*

*ROM1*

*RP1*

*RPE65*

*RPGRIP1*

*RPGRIP1L*

*SAG*

*SPATA7*

*TMEM216*

*TPP1*

*TRIM32*

*TRIM37*

*TTC8*

*TTPA*

*TULP1*

*USH1C*

*USH1G*

*USH2A*

*ZNF513*
